# Supplementary material for: A physical model describing the interaction of nuclear transport receptors with FG nucleoporin domain assemblies
Source: eLife. 2016 Apr 8;5:e14119. doi: 10.7554/eLife.14119 (PMC4874776; doi:10.7554/eLife.14119)
Supplement: Table 1—source data 1. — FG domains are shown in black letters, His tags in blue letters, and remaining parts (i.e., TEV cleavage sites, Cys tags and spacers) in grey letters. FxFG motifs are marked in yellow, GLFG motifs in green, other FG motifs in purple. Nup98-glyco features O-GlcNAc on ~30 of the S and T residues. DOI: http://dx.doi.org/10.7554/eLife.14119.004 [file elife-14119-table1-data1.zip › Table_1_source_data_1.pdf]

Table 1 – Source data 1. Amino acid sequence of employed FG domain constructs. FG domains are shown in black letters, His tags in blue letters, and remaining parts (i.e., TEV cleavage sites, Cys tags and spacers) in grey letters. FxFG motifs are marked in yellow, GLFG motifs in green, other FG motifs in purple. Nup98-glyco features O-GlcNAc on ~30 of the S and T residues.

	1	MGCNFNTPQQNKTPFSFGTANNNSNTTNQNSSTGAGAFGTGQSTFGFNNSAPNNTNNANS
	61	SITPAFGSNNTGNTAFGNSNPTSNVFGSNNSTTNTFGSNSAGTSLFGSSSAQQTKSNGTA
	121	GGNTFGSSSLFNNSTNSNTTKPAFGGLNFGGGNNTTPSSTGNANTSNNLFGATANANKPA
	181	FSFGATTNDDKKTEPDKPAFSFNSSVGNKTDAQAPTTGFSFGSQLGGNKTVNEAAKPSLS
	241	FGSGSAGANPAGASQPEPTTNEPAKPALSFGTATSDNKTTNTTPSFSFGAKSDENKAGAT
Nsp1	301	SKPAFSFGAKPEEKKDDNSSKPAFSFGAKSNEDKQDGTAKPAFSFGAKPAEKNNNETSKP
	361	AFSFGAKSDEKKDGDASKPAFSFGAKPDENKASATSKPAFSFGAKPEEKKDDNSSKPAFS
	421	FGAKSNEDKQDGTAKPAFSFGAKPAEKNNNETSKPAFSFGAKSDEKKDGDASKPAFSFGA
	481	KSDEKKDSDSSKPAFSFGTKSNEKKDSGSSKPAFSFGAKPDEKKNDEVSKPAFSFGAKAN
	541	EKKESDESKSAFSFGSKPTGKEEGDGAKAAISFGAKPEEQKSSDTSKPAFTFGAQKDNEK
		KTEGSSGSGSGGSGRSHHHHHHHHHH
	1	MSKHHHHSGHHHTGHHHHSGSHHHTGENLYFQGTSFNKTFGSPFGTGNGAFGATSTFGQT
	61	TGFGTTPATAFGSAGFGTNTSTGGLFGNTQTKPGGLFGSTTFNQPATSSSSSGFGFGAST
	121	GTTNSLFGSTNTGSGLFATQSNAFGQAKPTTFGNFGTSTSTGGLFGNTNTANPFGGTSAS
	181	LFGASTFSAAPTGTTIKFNPPSGTDTMAKGGVTTNISTKHQCITAMKEYESKSLEELRLE
Nup98-glyco	241	DYQANRKGPQNPVGAPTGTGLFGTSAATSSASTGIFGSTAANNSFSFAGNKTTFGTAGTG
	301	AFGGNTGGLFGQPANQPAASLFNKPFGNATTTQSTGFSFGNTSTLGQPQTSTMGLFGANQ
	361	PTQSGGLFGTTTNTNATGAFGAGTSLFGQPNPAPFGTGSTLFGNKPAGFGTTTTSAPAFG
	421	TTTGGLFGNKPTLTLGTNTNTSNFGFGSNTAGTSLFGNKTATGTIGPSLGTGFGTALNPG
	481	QTSLFGSNQPKLTGTLGTGAFGNAGFNSTSAGLGFGAPQC
	1	MSKHHHHSGHHHTGHHHHSGSHHHTGENLYFQGSASTPAFSFGASNNNSTNNGTSTPAFS
	61	FGASNNNSTNNGTSTPAFSFGASNNNSTNNGTSTPAFSFGASNNNSTNNGTSTPAFSFGA
reg-FSFG	121	SNNNSTNNGTSTPAFSFGASNNNSTNNGTSTPAFSFGASNNNSTNNGTSTPAFSFGASNN
	181	NSTNNGTSTPAFSFGASNNNSTNNGTSTPAFSFGASNNNSTNNGTSTPAFSFGASNNNST
	241	NNGTSTPAFSFGASNNNSTNNGTSTPAFSFGASNNNSTNNGTSTPAFSFGASNNNSTNNG
	301	TSTPAFSFGASNNNSTNNGTSTPAFSFGASNNNSTNNGTSC
